# High-throughput assays to assess variant effects on disease

**DOI:** 10.1242/dmm.050573

**Published:** 2024-06-28

**Authors:** Kaiyue Ma, Logan O. Gauthier, Frances Cheung, Shushu Huang, Monkol Lek

**Affiliations:** Department of Genetics, Yale School of Medicine, New Haven, CT 06510, USA

**Keywords:** Deep mutational scanning, High-throughput functional assays, Variant interpretation

## Abstract

Interpreting the wealth of rare genetic variants discovered in population-scale sequencing efforts and deciphering their associations with human health and disease present a critical challenge due to the lack of sufficient clinical case reports. One promising avenue to overcome this problem is deep mutational scanning (DMS), a method of introducing and evaluating large-scale genetic variants in model cell lines. DMS allows unbiased investigation of variants, including those that are not found in clinical reports, thus improving rare disease diagnostics. Currently, the main obstacle limiting the full potential of DMS is the availability of functional assays that are specific to disease mechanisms. Thus, we explore high-throughput functional methodologies suitable to examine broad disease mechanisms. We specifically focus on methods that do not require robotics or automation but instead use well-designed molecular tools to transform biological mechanisms into easily detectable signals, such as cell survival rate, fluorescence or drug resistance. Here, we aim to bridge the gap between disease-relevant assays and their integration into the DMS framework.

## Teaser

**Summary:** The lack of diverse high-throughput functional assays for deep mutational scanning impedes genetic variant interpretation in rare disease research. This article reviews applicable assays across various molecular mechanisms.

**Figure:**
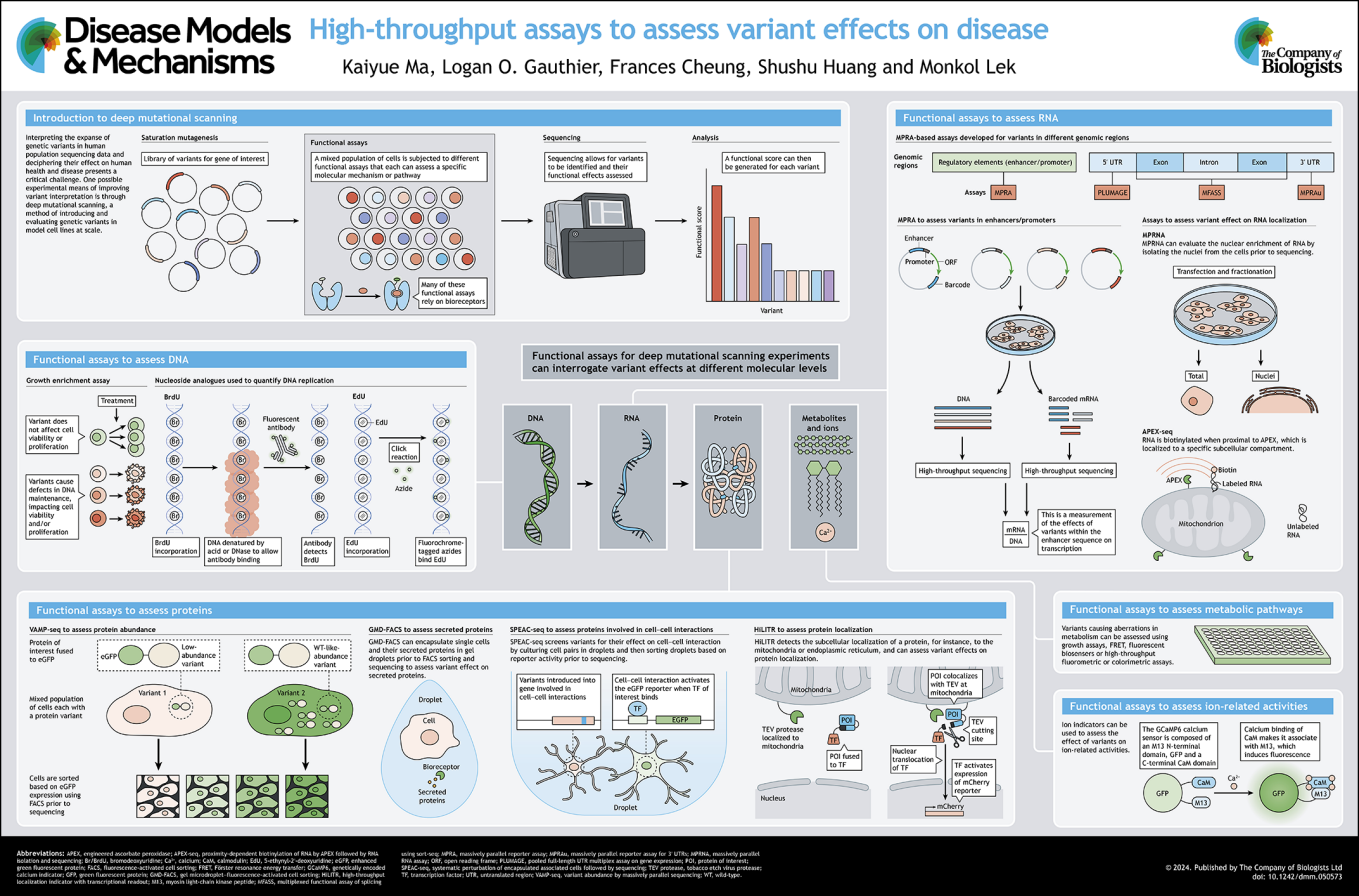
See supplementary information for a high-resolution version of the poster.

## Introduction

Rare diseases encompass a diverse group of disorders, often characterized by their low prevalence and complex etiology. Potential treatment requires an initial diagnosis; however, the diagnosis of rare genetic diseases presents significant challenges in clinical practice. The process of obtaining an accurate diagnosis for patients with rare disease typically takes an average of 4-5 years, with many individuals remaining undiagnosed (Hatirnaz Ng et al., 2022; Marwaha et al., 2022; Schaaf et al., 2020). Understanding and addressing the challenges of rare disease diagnostics are essential for advancing diagnostic practices and improving patient outcomes. Current sequencing technologies have enabled detection of numerous types of variants in the human genome (Mastrorosa et al., 2023; Satam et al., 2023). Although millions of genetic variants have been uncovered, progress in our ability to understand how these variants affect molecular, cellular and organismal phenotypes – known as the genotype–phenotype relationship – trails behind (Uffelmann et al., 2021). As a result, the comparatively underdeveloped state of clinical variant interpretation limits the potential to advance disease diagnostics and therapeutics (Hoffman-Andrews, 2017).

Thus far, variant interpretation studies have been driven predominantly by analyzing large reference population datasets. Based on known estimated mutation rates, every possible single nucleotide variant (SNV) that lacks severe pathogenic effects should theoretically exist in at least one living person (Gudmundsson et al., 2022; Johnston et al., 2019). However, human variants remain largely unclassified and, among the small portion that has been classified, roughly half are variants of uncertain significance (Henrie et al., 2018; Pir et al., 2022; Starita et al., 2017). This is primarily a consequence of the lack of sufficient case reports from population studies, especially in the context of rare diseases. Additionally, population data indicate that rare pathogenic variants can exist in healthy individuals, further complicating interpretation of rare variants based solely on their frequency in the general population (Xiang et al., 2020).

To address the challenges in clinical interpretation of genetic variants, four strategies have been used to collect evidence aiding in their classification: the use of segregation analysis in families with affected members; data sharing through ClinVar and other variant databases; computational pathogenicity predictions; and functional assays (Starita et al., 2017). Conventional variant interpretation operates in an after-the-fact manner, initially identifying variants within patient families or patient databases and subsequently conducting disease-relevant functional assays for these identified variants. However, this workflow is typically labor intensive and slow, and lacks robust reproducibility across different experiments and laboratories. On the other hand, the method of computational prediction has proven scalable and cost effective, with the potential to interpret and predict functional impact of all possible variants (Fowler et al., 2023; Livesey and Marsh, 2022). A wealth of computational methods has been developed recently with rapidly improving sensitivity and specificity (Cheng et al., 2023; Zhou et al., 2022). In addition to computational methods, functional assays also contribute to the rigor required by clinical diagnostics, which necessitate multiple lines of evidence. Functional assays are particularly powerful when employed in deep mutational scanning (DMS), also known as multiplexed assays for variant effects (MAVE), in which large variant pools are functionally characterized in a massively parallel manner (see poster) (Wei and Li, 2023).

A DMS workflow typically begins with creating a library of genetic variants and introducing them into an appropriate cell line, followed by the use of a reporter or signal system (Box 2) to monitor and measure the effects of the genetic variants on a specific cellular process or phenotype (see poster, ‘Introduction to deep mutational scanning’) (Findlay et al., 2018). Based on the reporter signal, cells are then separated and/or selected for those containing potential genetic variants that result in the expected phenotypes, after which sequencing determines the presence and abundance of each genetic variant (Weile and Roth, 2018). Lastly, conducting data processing, scoring and analysis allows assessment of the functional impact of each variant and identifies potential candidates of interest (Esposito et al., 2019; Starita and Fields, 2015; Tareen et al., 2022).

A variety of sophisticated reviews discuss the topic of variant interpretation, many of which offer a broad overview of DMS workflows and their role in computational prediction, or focus on small-scale functional assays for particular diseases such as cancers and specific molecules such as proteins (Fowler and Fields, 2014; Gasperini et al., 2016; Gelman et al., 2019; Livesey and Marsh, 2022; Weile and Roth, 2018). Other reviews also highlight the importance of choosing functional assays based on specific disease mechanisms, either introducing approaches with high clinical value (Findlay, 2021) or discussing assays for variant effects on gene regulation, protein function and cellular phenotype as part of a broad discussion of multiplexed sequence–function workflow (Tabet et al., 2022). Building upon these foundations, this At a Glance article takes a comprehensive perspective on genetic disease mechanisms that span molecular levels in depth, from DNA and RNA to proteins and their subsequent functions (see poster). For protein functions, we have focused on ion handling and metabolites associated with ion channels and metabolic enzymes (see poster, ‘Functional assays to assess metabolic pathways’ and ‘Functional assays to assess ion-related activities’). Such an approach is uniquely conducive to the systematic study of rare disease variants for which the mechanistic impacts and the molecular level of their influence are often unknown. We seek to bridge the gap between disease-relevant assays and their integration into the DMS framework, offering detailed insights from existing studies and innovative proposals for the high-throughput application of these assays in diverse rare genetic disease contexts. Overall, this At a Glance article serves as a contribution to collaborative efforts in creating an Atlas of Variant Effects (Fowler et al., 2023).

**Box 1. Glossary**

**Aptamers:** a type of antibody mimetics, including short polynucleotides or peptides that can be synthetically generated and can bind a specific target molecule.

**Bioreceptors:** biological or biomimetic components that recognize the target analyte and produce measurable signals that are proportional to the concentration of the analyte.

**Click chemistry:** a category of simple, atom-economy reactions for joining two desired molecular entities. This methodology enables the conjugation of fluorophores and other reporter molecules with biomolecular probes.

**Fluorescence flow cytometry:** a high-throughput quantitative assay suitable for a wide range of bioreceptors. As cell populations flow past lasers, detectors are used to rapidly analyze cells based on fluorescence signals.

**Förster/fluorescence resonance energy transfer (FRET):** a mechanism in which energy is transferred between a donor and an acceptor chromophore. The efficiency of this transfer decreases as the distance between them increases. Consequently, FRET can serve as a fluorescence indicator to detect a specific ligand that brings the FRET pair into close proximity.

**Nanopore:** a pore of nanometer size, which has recently seen increasing use in the sequencing of biopolymers, including DNA, RNA and proteins.

**Saturation mutagenesis**: the process to introduce all possible variants, mostly single nucleotide variants or single amino acid substitutions in the entire sequence of a given gene or genomic region, which can be achieved either exogenously or endogenously.

**Untranslated region (UTR):** there is one UTR on each end of a mRNA. The 5ʹ UTR is known as the leader sequence and the 3ʹ UTR is known as the trailer sequence.

**Box 2. A highlight for bioreceptor-based assays**

This At a Glance article presents illustrative examples of high-throughput assays, many of which use bioreceptors (Box 1). Bioreceptor-based assays are a critical component of deep mutational scanning, and recent advancements in bioreceptor design have facilitated greater efficacy and easier production. Considering the generalizability of these assays, researchers interested in performing deep mutational scanning are recommended to search for available bioreceptors or design their own before considering alternative assay methods. Historically, antibodies have been the most widely used bioreceptors, as their remarkable versatility allows them to bind specifically to a wide variety of antigens, including proteins (see poster, ‘Functional assays to assess proteins’), DNAs (see poster, ‘Functional assays to assess DNA’), RNAs (see poster, ‘Functional assays to assess RNA’), lipids and sugars (Alving, 2006; Doerr, 2008; Kappler and Hennet, 2020; Wang and Xia, 2019). Notably, haptens (small molecules less than 1 kDa in size), such as the neurotransmitters serotonin and dopamine, can also be recognized by corresponding antibodies when attached to larger protein carriers, such as albumins, thyroglobulins, hemocyanins and polylysine (Clementi et al., 1991; Huisman et al., 2010). Beyond traditional antibodies, newly developed bioreceptors offer simplified designs and manufacturing processes (Crivianu-Gaita and Thompson, 2016). Fragment antibodies, such as single-chain variable fragments (scFvs), contain only the functional regions of antibodies, increasing their specificity and penetrability (Ahmad et al., 2012). Aptamers (Box 1), including oligonucleotides and short peptides, can be programmatically designed and manufactured to selectively bind to specific targets (Adachi and Nakamura, 2019; Reverdatto et al., 2015). This design flexibility arises from their standardized composition, such as the four nucleotides in DNA aptamers. Consequently, aptamers can undergo directed evolution, enabling the rapid generation of new aptamers tailored to specific targets. The most commonly used method for selecting specific aptamers is the Systematic Evolution of Ligands by EXponential Enrichment (SELEX), which involves screening a library of random sequences to identify aptamers for a desired target (Liu et al., 2020). Other small-molecule tools include ligand-dependent transcription factors and riboswitches, as well as Förster resonance energy transfer (FRET) systems (Box 1) that generate a fluorescence signal when two molecules bind together (Liu et al., 2017).

## Assays for diseases related to DNA replication, repair or modification

Disease mechanisms that disrupt biochemical properties of DNA can have catastrophic effects on human health, necessitating effective assays to evaluate variants underlying diseases in DNA replication, repair and modification pathways. These variants can act via *in situ* effects specific to the mutational sequence that alters DNA modifications or replication origin motifs, or by deactivating complex machinery involved in repair systems. The consequences of malfunctions in DNA replication and repair, which are essential for development and homeostasis, include congenital disorders such as Meier–Gorlin syndrome and accelerated aging disorders, whereas disruptions to DNA modifications critical for expression regulation, transposon silencing and DNA repair are associated with imprinting disorders (Bellelli and Boulton, 2021; Bicknell et al., 2011; Liyanage et al., 2014; Robertson, 2005; Tiwari and Wilson, 2019).

Due to the essential role of DNA integrity in cell function, pathogenic genetic variants in associated pathways often diminish cell viability. Cell growth enrichments thus serve as simple high-throughput assays for evaluating variants, as variants negatively impacting cell viability or proliferation are gradually eliminated, whereas variants with neutral functional impact become enriched (see poster, ‘Functional assays to assess DNA’). The efficacy of such assays has been demonstrated in DMS studies on the impact of genetic variants on DNA mismatch repair (MMR). MMR detects and repairs sequence errors on newly synthesized strands during DNA replication, recombination or damage repair (Iyer et al., 2006), and loss-of-function variants of the MMR gene *MSH2* are associated with Lynch syndrome, a hereditary predisposition to cancers (particularly colorectal cancer). In a recent study of *MSH2*, the purine analog 6-thioguanine (6-TG) was used to activate the MMR system and induce cell death upon its incorporation into DNA (Jia et al., 2021). Loss-of-function variants in *MSH2* resulted in defective MMR, causing resistance to 6-TG toxicity. This enabled the application of cell growth enrichment to selectively identify these deleterious variants. This assay has also been adapted for other MMR-related genes, including *MSH6* and *MLH1* (Frederiksen et al., 2021; Yan et al., 2003). To enhance the lethality and strength of selection in these growth enrichment assays, genetic predisposition can be induced by introducing variants in related genes in the cell lines used before performing the assay. For instance, knockout of the DNA repair gene *PARP14* allowed identification of genes essential for the viability of PARP14-deficient cells (Dhoonmoon et al., 2020).

In addition to cell growth enrichment assays, DNA replication and damage can also be directly detected using fluorescent reporters, for which assays have yet to be established in DMS studies. The nucleoside analogues bromodeoxyuridine (BrdU) and 5-ethynyl-2ʹ-deoxyuridine (EdU) are commonly used to quantify DNA replication, during which their incorporation into nascent DNA generates detectable signals (see poster, ‘Functional assays to assess DNA’). This can potentially be incorporated into a DMS workflow to identify variants that disrupt DNA repair mechanisms. BrdU detection is antibody based and, in addition to quantifying DNA replication, it has also been used to quantify DNA damage by labeling double-strand breaks (Konishi et al., 2011; Li and Darzynkiewicz, 1995). Conversely, EdU detection utilizes ‘click chemistry’ (see Glossary, Box 1) to conjugate fluorochrome-tagged azides with EdU molecules (Krishan and Hamelik, 2010). As DNA-incorporated EdU can be removed by the DNA repair mechanism of nucleotide excision repair (NER) (Wang et al., 2022), EdU-based assays also have the potential to assist variant interpretation for NER-associated diseases. For instance, xeroderma pigmentosum, a condition marked by heightened sensitivity to ultraviolet (UV) light, is linked to variants in nine genes within the NER pathway. Additionally, Cockayne syndrome, a developmental disorder with a significant impact on lifespan, is associated with variants in two NER pathway genes (Cleaver et al., 2009). Thus, the use of BrdU and EdU fluorescent reporter assays in DMS studies can enable further study of variants in these genes.

Various alternative strategies exist for the detection of DNA damage and have further facilitated high-throughput variant interpretation, but these have yet to be incorporated in DMS studies. For instance, flow cytometry-based nucleoside analogue assays have been developed and used to evaluate DNA damage responses triggered by UV light or the DNA topoisomerase I inhibitor topotecan (Darzynkiewicz et al., 2011). Additionally, several methods have emerged for cytometric detection of DNA damage by utilizing nucleic acid-binding dyes such as Acridine Orange or by targeting factors recruited to strand breakpoints (Huang et al., 2005). These assays have been used to identify apoptotic cells from a pool and may be adapted to quantify variant exclusion in non-apoptotic cells in a DMS setting. High-throughput microscopy and array-based assays have also been used to evaluate DNA damage (Gallant et al., 2022; Sykora et al., 2018). Incorporating these assays into a DMS workflow could further expand interpretation capabilities for variants in genes associated with DNA replication or repair.

In summary, several methods including growth-based assays and flow cytometry-based assays can be employed to study variant effects on DNA molecules. Characterizing these variants using DMS can aid in improving clinical genetic testing for severe rare diseases rooted in DNA replication, repair and modification pathways.

## Assays for diseases related to RNA transcription, splicing or localization

As RNA serves to convert the genetic information encoded in DNA into biological functions executed by RNA or proteins, variants in genes regulating processes such as RNA transcription, splicing and localization are associated with a multitude of disorders, including rare diseases such as Opitz–Kaveggia syndrome and retinitis pigmentosa (Lee and Young, 2013; Scotti and Swanson, 2016). Recently, researchers have recognized the importance of characterizing these previously overlooked variants to aid in resolving rare disease cases (Frésard et al., 2019; Montgomery et al., 2022).

To evaluate variants associated with transcriptional misregulation disorders, high-throughput characterization of transcription regulation is achieved using reporter assays via endogenous knock-in or exogenous reporter constructs (see poster, ‘Functional assays to assess RNA’) (Diao et al., 2017; Lee and Young, 2013; Rajagopal et al., 2016). The endogenous knock-in preserves the correct genomic context and hence may better reveal the *in vivo* regulation landscape. Assays using exogenous reporter constructs lose the genomic context but can be easily performed on a larger scale for regulatory elements of multiple genes. One frequently used assay that uses exogenous reporter constructs is the massively parallel reporter assay (MPRA), in which variants are introduced to their respective constructs and characterized in parallel according to the reporter signals (e.g. the enrichment of a sequencing tag) (Melnikov et al., 2012). Such MPRA methods have been successfully established in previous DMS studies. In the Melnikov et al. (2012) study, each construct contained an enhancer with a single-hit variant or multi-hit variants, an invariant promoter, a luciferase gene and a unique sequencing tag used to identify the variant(s) for the MPRA. Similarly, in an MPRA study evaluating schizophrenia- and Alzheimer's disease-associated variants that were hypothesized to affect gene transcription, each construct carried a genome-wide association study (GWAS)-associated *cis*-regulatory variant, a minimal promoter, a green fluorescent protein (GFP) and a sequencing tag that was used to quantify variant enrichment (Myint et al., 2020). In both studies, variant effects on gene expression were assessed through the enrichment of sequencing tags rather than the signal strength of the luciferase/GFP, as a single cell could contain multiple constructs, thus reporter signal would not directly reflect enhancer activity. Site-specific integration, in which each cell is controlled to carry only one construct, was used in an MPRA for identification of mammalian enhancers (Dickel et al., 2014). In this study, each construct contained a sheared genomic sequence (∼1-1.6 kb) that was evaluated for enhancer activity, a minimal promoter and a gene encoding a yellow fluorescent protein (YFP), used to quantify enhancer activity. This strategy made it possible to perform MPRA using a fluorescence flow cytometry assay (Box 1).

Similar to transcriptional misregulation, variants altering post-transcriptional regulation of RNA splicing and stability are linked to severe rare diseases, including spliceosomopathies and RNA degradation disorders (Griffin and Saint-Jeannet, 2020; Weskamp and Barmada, 2018). MPRA-based methods have been adapted to examine post-transcriptional regulations. To study splicing, variants can be introduced into an artificial region inserted in a canonical exon or canonical intron, and different splicing events in this region may switch on or switch off one reporter [multiplexed functional assay of splicing using sort-seq (MFASS)] (Cheung et al., 2019) or two reporters in different frames (Stork and Zheng, 2018). MPRA-based assays can also assess the functional effects of variants located in the 5ʹ untranslated region (UTR) and 3ʹ UTR (Box 1), which are involved in many gene expression regulation mechanisms, including RNA stability, RNA localization and translation initiation (Leppek et al., 2018; Mayr, 2019). For instance, employing pooled full-length UTR multiplex assay on gene expression (PLUMAGE) identified functional 5ʹ UTR SNVs in prostate cancer (Lim et al., 2021), whereas MPRA for 3ʹ UTRs (MPRAu) identified variants that regulate the viral defense gene *TRIM14* and modify *PILRB* expression (Griesemer et al., 2021).

Proper RNA function also relies on its localization, and modification of MPRA-based methods to quantify enrichment of RNA in subcellular compartments has enabled their application in studying RNA mislocalization-related diseases. The incorporation of such assays in DMS studies could thus enhance interpretation of RNA localization-related variants in these diseases. For instance, amyotrophic lateral sclerosis (ALS), a disease characterized by reduced muscle functionality and nerve cell breakdown, is due to disruptions in the transport of RNA to distal parts of neurites (Basyuk et al., 2021). This perturbation is rooted in variants in *ANXA11*, which encodes annexin proteins that connect RNA granules to lysosomal membranes. As RNA from soma and neurite compartments in neurons can be collected separately, variants that affect RNA transport, such as those in *ANXA11*, can be identified (Mikl et al., 2022). Modified MPRA methods similarly enable study of nuclear-retained long non-coding RNAs (lncRNAs), key regulators of gene expression that have functions that depend on subcellular localization. Massively parallel RNA assay (MPRNA) can evaluate the nuclear enrichment of RNA by isolating the nuclei from the cells (see poster, ‘Functional assays to assess RNA’), revealing unique nuclear localization domains of lncRNAs, such as *MALAT1*, which facilitates transcriptional activation and is associated with cancer metastasis (Shukla et al., 2018; Sun et al., 2018). Although these assays require the often-complex isolation of RNAs from different subcellular compartments, proximity-labeling techniques, such as APEX-seq, have demonstrated promise as an alternative universal assay for RNA localization (see poster, ‘Functional assays to assess RNA’). In APEX-seq, RNA molecules within a few nanometers of APEX2, a peroxidase enzyme that is genetically targeted to the cellular region of interest, are biotinylated. The biotinylated RNA can then be isolated using streptavidin-coated beads without requiring separate collection of subcellular compartments and sequenced (Fazal et al., 2019).

Thus, MPRA and its derived methods are useful in characterizing variants affecting transcriptional and post-transcriptional regulations, whereas proximity-labeling techniques possess high potential for studying RNA localization. These methods can help identify undiscovered rare disease variants in genes underlying RNA processes.

## Assays for diseases related to protein abundance or misfolding

Considering the role of proteins as the chief enactors of cellular functions, variants that diminish protein abundance or impede proper folding can cause severe rare diseases. Often rooted in amino acid substitutions with significantly different biochemical properties, damaging missense variants can contribute to decreased translation efficiency or instability associated with translation deregulation disorders (Tahmasebi et al., 2018). Missense variants can also cause misfolding, resulting in proteinopathies (Hanna et al., 2019) that comprise a large variety of diseases, most notably, cystic fibrosis and Alzheimer's disease (Ashraf et al., 2014; Meng et al., 2019). Whether due to decreased translation or structural defects, the overall reduction of functional protein levels underlies the majority of haploinsufficient diseases (Bartha et al., 2018; Birolo et al., 2021; Deutschbauer et al., 2005).

One universal assay to screen variants affecting protein abundance is variant abundance by massively parallel sequencing (VAMP-seq), which was been successfully employed in DMS studies (see poster, ‘Functional assays to assess proteins’). VAMP-seq utilizes a pool of plasmids expressing variants in a gene of interest fused to eGFP, which is integrated into cells through recombination such that variants that reduce target protein abundance should result in lower fluorescence signal (Matreyek et al., 2018). Matreyek et al. (2018) applied VAMP-seq to evaluate single amino acid variants of *PTEN*, a tumor suppressor gene, and showed ‘selection for low-abundance *PTEN* variants’ as a common oncogenic mechanism. Variants were also screened in *TPMT*, which encodes thiopurine methyltransferase. TPMT enzyme activity dictates the toxicity of thiopurine drugs used to treat acute lymphoblastic leukemia, suggesting the therapeutic implications of such assays. Furthermore, it has been inferred that using C-terminally tagged eGFP in addition to regular VAMP-seq may allow the assessment of the effects of variants on protein conformation (Chiasson et al., 2020). The main limitation of VAMP-seq is its reliance on eGFP, which can be overcome using small epitope tags such as FLAG accompanied with their corresponding antibodies. The general VAMP-seq framework is thus a simple yet powerful tool to study a broad range of disease mechanisms rooted in abnormal protein abundance.

Secreted proteins function outside the cell membrane, meaning that they disassociate from the cells that produced them, making them unsuitable for DMS assays such as VAMP-seq that rely on isolating cellular DNA that encodes the corresponding mutant proteins (Chiasson et al., 2019). Nevertheless, evaluating secreted protein abundance is vital to variant characterization as secreted proteins mediate cell communication and perturbations in the secretome are implicated in the pathogenesis of metabolic disorders, cancer and neurodegenerative diseases (Song et al., 2019). The method of gel microdroplet–fluorescence-activated cell sorting (GMD-FACS) can be adapted to address this issue by encapsulating single cells and their secreted proteins in gel microdroplets (see poster, ‘Functional assays to assess proteins’) (Fang et al., 2017). Fang et al. (2017) used GMD-FACS to link individual yeast cells with the monoclonal antibodies they secreted, demonstrating the efficacy of the assay in assessing variant impact on corresponding secreted protein levels and its potential for novel use in DMS studies.

Detecting protein malfunction is further complicated for proteins that rely on interactions with other molecules, such as in cell–cell interactions in the central nervous system that are implicated in neurological diseases (Linnerbauer et al., 2020; Sanmarco et al., 2021). This necessitates assays capable of screening intercellular mechanisms. Systematic perturbation of encapsulated associated cells followed by sequencing (SPEAC-seq) was developed as a droplet-based high-throughput platform enabling forward genetic screens of cell–cell interaction mechanisms (see poster, ‘Functional assays to assess proteins’) (Wheeler et al., 2023). This assay involves co-encapsulation and co-culturing of two cells within droplets, allowing them to remain isolated from neighboring cell pairs, while each pair interacts through direct contact and/or exchange of secreted soluble factors. Upon successful interaction, the cell pair exhibits activation of a reporter, such as eGFP, that can be detected through droplet sorting. This demonstrates the potential of high-throughput SPEAC-seq to be incorporated in DMS studies to evaluate gene variants involved in cell–cell interaction.

In summary, the use of VAMP-seq to study variant effects on intracellular protein abundance can be further enhanced by improving tagging strategy, whereas the study of extracellular proteins or cell–cell interactions can be facilitated by droplet-based methods. These can further expand the scope of DMS in rare disease studies by allowing detection of variants disrupting protein folding and abundance.

## Assays for diseases related to protein modification or localization

Beyond abundance, proper post-translational modifications (PTMs) and translocalization of existing proteins is crucial in enabling rapid and efficient responses to cellular changes, which would otherwise be unattainable by energy-expensive and time-consuming protein synthesis. Variants affecting protein modifications such as acetylation, glycosylation and methylation have been implicated in various rare disease etiologies, such as dystroglycanopathy disorders that result from hypoglycosylation of α-dystroglycan (α-DG, encoded by *DAG1*) (Bauer et al., 2015; Kanagawa, 2021; Xu et al., 2018). For DMS studies on PTMs, methods relying on bioreceptors (Boxes 1 and 2) are prioritized due to their compatibility with pooled saturation mutagenesis (Box 1). For example, IIH6C4 is an antibody specific to glycosylated α-DG that can be coupled to flow cytometry (Stevens et al., 2013). Although this cytometric assay was developed to evaluate α-DG glycosylation in fibroblasts from patients with variants in associated enzymes, it can be easily adapted for DMS studies to improve functional variant interpretation for more than a dozen enzymes involved in α-DG glycosylation (Ma et al., 2023 preprint). Additionally, nanopore (Box 1) technologies provide an alternative and more generalizable method for detecting different PTMs based on their distinctive ionic current signals (Restrepo–Pérez et al., 2019). This can potentially be developed into high-throughput assays by fusing target proteins with unique barcode peptides, allowing nanopores to identify the PTMs on the target proteins and link them to specific phenotypes (Afshar Bakshloo et al., 2022).

Because protein function often relies on localization in designated compartments, protein mislocalization can also generate disease via protein inactivation or toxic misregulation (Owji et al., 2018). For example, mislocalization of the AGT enzyme from peroxisomes to the mitochondria causes primary hyperoxaluria type 1, a rare and progressive kidney disease (Hung and Link, 2011). The effects of such mislocalizations can be studied using assays such as high-throughput localization indicator with transcriptional readout (HiLITR), which converts protein localization into detectable fluorescence signals (see poster, ‘Functional assays to assess proteins’) (Coukos et al., 2021). In HiLITR, a tobacco etch virus (TEV) protease is programmed to localize at a given site, such as the mitochondrial or endoplasmic reticulum (ER) surface, and a transcription factor (TF) is fused to the protein of interest (POI) via a linker containing a TEV cutting site. Upon colocalization of the POI and the TEV, the TF is released, it translocates to the nucleus and activates the expression of a fluorescence reporter. Via HiLITR, Coukos et al. (2021) discovered that *SAE1* and *EMC10* were associated with mislocalization of mitochondrial and ER-anchored proteins, demonstrating the potential for HiLITR to detect subcellular protein localization-related variants in DMS studies. In addition, HiLITR has the potential to be adapted to study variant effects on other subcellular localizations, such as the mitochondrial matrix. In this scenario, rather than POIs being fused to a TF, they can be fused to a dark-to-bright protease reporter carrying the TEV cutting site, such as FlipGFP (Zhang et al., 2019) or cyclic mNeonGreen2 (Gong et al., 2022). Then, a mitochondrial targeting sequence can be added to the TEV protease to reveal altered intramitochondrial localization of the POI–reporter fusion protein. In addition to HiLITR, employing high-throughput microscopy enhanced with deep learning can identify several specific subcellular compartments and assess protein localization. This was successfully demonstrated in a study that accurately classified protein localization for 12 subcellular compartments (Pärnamaa and Parts, 2017).

Thus, although bioreceptors are currently the most applicable tools for assessing PTMs, nanopore technologies are being rapidly developed for this purpose. In addition, HiLITR-based assays hold the potential for DMS applications characterizing subcellular protein localizations. Such assays can therefore facilitate discovery of variants impacting protein modification or localization in rare diseases.

## Assays for diseases related to metabolic pathways and metabolites

Although functional protein activity is required for crucial cellular operations such as metabolism, the small-molecule metabolites produced during these processes also influence cell viability. Variants causing aberrations in metabolic pathways or metabolites can disrupt nutrient use and biosynthesis or accumulate toxicity, leading to a wide range of rare, inherited metabolic disorders such as phenylketonuria and Tay–Sachs disease (Hoffmann et al., 2002). Growth assays have been developed in efforts to characterize variants associated with these metabolites (see poster, ‘Functional assays to assess metabolic pathways’). One common strategy employs yeast surrogate platforms, in which a yeast gene is replaced with the human ortholog carrying the variant of interest. This method has been used in a DMS study to assess the functional effects of over 1500 SNV-accessible single amino acid substitutions in the human *OTC* gene, which encodes ornithine transcarbamylase and is linked to the most prevalent urea cycle disorder (Lo et al., 2023). In this study, the human *OTC* gene was incorporated into a yeast strain lacking the yeast ortholog *ARG3*, and as *ARG3*/*OTC* is required for yeast to grow on an arginine-deficient medium, *OTC* variants depleted from the arginine-deficient growth assay could be identified as likely pathogenic variants. However, Lo et al. (2023) reported a discordance between the yeast and human cellular contexts. Such cross-species discrepancy can be avoided by designing and performing growth assays in human cell lines, as demonstrated by the glucose-galactose assay used for studying mitochondrial metabolism (Sanuki et al., 2017). As fast-growing cells mainly rely on glycolysis, they are unaffected by mitochondrial impairment in glucose-containing medium. Conversely, galactose-containing medium forces cells to shift from glycolysis to oxidative phosphorylation, increasing their sensitivity to mitochondrial impairment. The glucose-galactose assay thus has the potential for novel use to evaluate variants in the mitochondrial genome in human cells (Arroyo et al., 2016). Nevertheless, both yeast and human cell platforms may generate results discrepant from *in vivo* variant effects and, thus, validation with known benign/pathogenic variants is essential for any assay.

Apart from growth assays, bioreceptor-dependent methods offer alternative, often preferred strategies to assess variants impacting metabolites. As ATP is the primary cellular energy carrier and a universal kinase substrate, ATP levels reflect generalized kinase functionalities. In a flow cytometry-based assay for ATP, a Förster resonance energy transfer (FRET) system (Box 1) was used to generate ATP-dependent signals, which consisted of a modified cyan fluorescent protein and a monomeric Venus fluorescent protein separated by an ATP-binding domain (Mendelsohn et al., 2018). This assay enabled identification of genes encoding ATP-dependent proteins, including *SLC30A9* and *SNRPD3* that were previously not known to be involved in energy metabolism, and revealed an enrichment in mitochondrial pathways. FRET flow cytometry has also been developed for glucose, the primary energy source in metabolism, which utilizes a FRET pair consisting of an enhanced cyan fluorescent protein and an mCitrine separated by a glucose-binding domain (Voyton et al., 2018). Another assay has been specifically designed for lipid droplets, which are crucial in energy supply and cellular homeostasis. In this assay, the fluorescent biosensor ‘C-Py’ was designed and synthesized to precisely target lipid droplets, which is compatible with flow cytometry (Zhang et al., 2021). The incorporation of such assays in DMS studies can therefore enable assessment of variants in ATP-, glucose- and lipid droplet-related genes.

In addition to the aforementioned methods, microtiter plate-based assays are available for a range of essential metabolites. Fluorometric assays are applicable to both ATP (Koresawa and Okabe, 2004) and lipids, whereas lipids can also be evaluated via colorimetric assays (Fulton et al., 2018) based on fluorescence quenching (Cho et al., 2017) or FRET (Niedziela-Majka et al., 2012). Colorimetric assays also enable sugar analysis (Gantt and Thorson, 2012), as does near-infrared spectroscopy (Srichan et al., 2022), which may also be adapted for high-throughput applications. A notable example of amino acid assessment is the enzyme cascade fluorescence-based assay, which quantifies the toxic accumulation of phenylalanine in phenylketonuria, an inborn error of metabolism caused by mutated phenylalanine hydroxylase (Flydal et al., 2019; Meng et al., 2022). The scale of these assays can be significantly increased for use in DMS studies by incorporating microdroplet approaches, enabling massively parallel reactions to occur within individual microdroplets (Mallires et al., 2020).

Thus, metabolic pathways play a significant role in rare disease mechanisms, with various metabolite-specific assays available such as growth, flow cytometry-based, microtiter plate-based and enzyme cascade fluorescence-based assays. Adapting these assays for DMS applications can aid in resolving genetic variants associated with rare metabolic diseases.

## Assays for diseases related to ion-related activities

Proper protein function and localization are also implicated in cellular regulation of ion concentrations and electric membrane potentials to support vital physiological processes, including development, regeneration and cell communication. For instance, mitochondria rely on ion gradients to generate energy, whereas organs such as the heart, brain and skeletal muscles function through action potentials (Adams and Levin, 2012). Disruptions to these processes produce conditions such as epilepsy, Parkinson's disease, autoimmune disorders such as multiple sclerosis, and various heart conditions (Djamgoz and Levin, 2020), illustrating the clinical significance of assessing the effects of variants that impact ion homeostasis.

Cardiovascular disorders such as genetic arrhythmias, atrial fibrillations and dilated cardiomyopathies can arise from variants in *SCN5A*, which encodes a major voltage-gated sodium cardiac channel. DMS using an adapted three-drug cytotoxicity assay has demonstrated promise in evaluating variant functional effects of *SCN5A* (Glazer et al., 2020). The cytotoxicity assay utilizes two sodium channel agonists, veratridine and brevetoxin, which induce sodium influx, as well as an Na^+^/K^+^ exchange inhibitor, ouabain, which increases sensitivity to sodium overload. Consequently, cells expressing functional sodium channels undergo apoptosis, and treating cells harboring *SCN5A* variants with the triple-drug mixture produces a graded response in cell survival, with gain-of-function and loss-of-function variants exhibiting decreased and increased viability, respectively.

Besides cytotoxicity assays, fluorescent ion indicators present a potential alternative for studying ion homeostasis and developing high-throughput screening assays. Indicators are categorized as small-molecule, genetically encoded or hybrid, which all utilize sensors consisting of a metal-binding group and at least one fluorophore (Carter et al., 2014). Small-molecule probes are exogenous compounds that either increase fluorescence intensity (‘intensity-based’) or shift excitation and/or emission wavelengths (‘ratiometric’). Genetically encoded sensors are fluorescent proteins attached to ion-binding proteins, such as Zn^2+^-binding proteins. Zn^2+^ is an essential structural constituent of proteins such as receptors, enzymes, TFs and growth factors, and approximately 10% of proteins functionally require Zn^2+^ binding. Deficiencies in Zn^2+^ binding can therefore elicit conditions such as immunodeficiencies, rare growth disorders and cancer progression (Fukada et al., 2011). Most genetically encoded Zn^2+^ sensors monitor Zn^2+^ levels via FRET efficiency, and although FRET-based sensors are preferred for quantifications, single fluorescent protein-based sensors have become popular for their greater dynamic ranges and ability to be multiplexed with other fluorescent sensors (Pratt et al., 2021). Such sensors have been developed and optimized for high-throughput screening of Ca^2+^ channels, demonstrating their potential for incorporation in DMS studies of ion channel-related variants. For example, Ca^2+^ binding of GCaMP6 induces fluorescence and this indicator can be coupled with a blasticidin selection marker via a self-cleaving peptide to allow the generation of stable-expressing 293-F clonal cell lines for intracellular Ca^2+^ assays (see poster, ‘Functional assays to assess ion-related activities’) (Wu et al., 2019). Finally, hybrid probes have both genetically encoded and exogenous components. One such system is the carbonic anhydrase platform, which can be utilized for sensing ion levels, particularly for Zn^2+^ and Cu^2+^ (Hurst et al., 2010; McCranor et al., 2012).

Studying electrophysiological characteristics also elucidates ionic activity, for which fluorescent bioelectricity reporters (FBRs) have proven viable (Adams and Levin, 2012). Fast-response FBRs respond to fluctuations in the electric field by undergoing an intramolecular change in electronic structure and consequently fluorescence properties. FBRs thus hold potential for high-throughput applications.

In summary, ion activities may underlie several rare disease mechanisms, from immunodeficiencies to growth disorders, and DMS-compatible assays such as growth assays and bioreceptor-based assays hold potential for broader use in research of such diseases.

## Challenges and opportunities

Recognizing the potential of DMS for interpreting rare variant effects, this At a Glance article offers a variety of context-specific functional assays capable of translating biological disease mechanisms at every molecular level into measurable indicators. This is an essential step in characterizing rare disease etiology, particularly considering the current lack of understanding of genetic variant effects in rare diseases and their consequently challenging diagnosis. Many of the discussed strategies use either enrichment culture assays separating variants by cell viability or flow cytometry assays segregating variants by fluorescence signals reflecting biological factors. These assays are compatible with pooled screens and thus facilitate high-throughput and cost-effective DMS workflows. An overview of numerous flow cytometry assays is presented, emphasizing the role of bioreceptors in converting biological activity into detectable fluorescence signals, as well as alternative assays for diseases without available bioreceptors or for validation purposes.

A variety of important factors require consideration when conducting functional assays for DMS. As many of the aforementioned assays have yet to be utilized in the DMS framework (Table 1) and some have yet to be established in high-throughput contexts, challenges may arise in effectively and reproducibly scaling up these assays. For example, although proximity-labeling techniques such as APEX-seq hold potential for RNA localization assays, scalability and reproducibility hinge on the optimization of fusion constructs in cells of interest (Fazal et al., 2019). Additionally, the use of FBRs in the detection of ionic fluctuations has been successful in small samples of cultured cells; however, their use has not yet been demonstrated on a larger scale. Although fluorescence imaging is amenable to large cell populations, the robustness and subcellular sensitivity of FBR signals in high-throughput, non-excitable cell contexts must be further optimized (Adams and Levin, 2012; Nikolaev et al., 2023). Evaluation of the challenge of scalability is discussed further in existing reviews (Livesey and Marsh, 2022; Tabet et al., 2022).

**Table 1.**
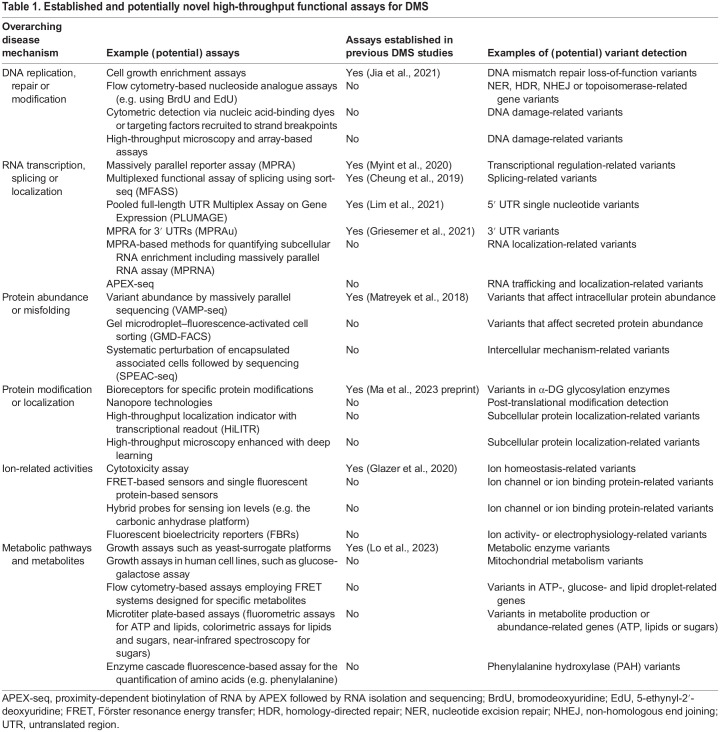
Established and potentially novel high-throughput functional assays for DMS

Additionally, many of the discussed assays were originally developed for purposes such as gene discovery, for which low-sensitivity assays can be sufficient. Such assays may therefore require further optimization and adaptation to achieve the sensitivity and dynamic range essential for distinguishing between pathogenic and benign variants. A significant challenge in selecting and interpreting functional assays also stems from the intricate relationship between protein function and disease pathogenicity, which raises several specific considerations. Firstly, the cell line platforms used in DMS may poorly resemble physiological events, as they may only reflect physiological functions in certain tissues and variant expression levels in these platforms may differ from physiological/pathological levels, thus reducing the sensitivity to separate pathogenic variants from benign variants. Secondly, the quantification of the functional impacts of variants may encounter complications because of potential feedback loops within cells. These loops might influence variant effects of different functional levels, therefore impacting the sensitivity of the DMS experiment and the accuracy of pathogenicity prediction (Marciano et al., 2016). Additionally, some variants impact multiple levels of molecular processes, such as a variant in the coding sequence causing disease mechanisms involving both RNA and protein. Thus, it may be necessary to assess both RNA and protein with relevant assays to accurately evaluate the functional effects of a variant. In summary, it is beneficial to employ different assays to characterize variants in the same gene when applicable. This not only provides orthogonal validation, but also has the potential to elucidate distinct disease mechanisms. The significance of integrating multiple functional assays to improve assessment of variant effects is discussed further by Findlay (2021).

Another crucial consideration lies in the meticulous interpretation of DMS findings, as both the experimental design and data analysis presume that the specific disease mechanism under investigation is solely responsible for the observed symptoms. This assumption does not always prevail, however, as many genes have multiple functions and different variants of these genes can cause entirely unrelated diseases through distinct pathological pathways (Gratten and Visscher, 2016). Similarly, it is essential to acknowledge that as DMS often focuses on a single aspect of gene function, it may conceal the pathogenicity of certain variants and lead to inaccurate variant classification. For example, *GNE* has two distinct enzymatic functions within the sialic acid biosynthesis pathway associated with its two functional domains (Carrillo et al., 2018; Huizing, 2005). Other genes may also have additional functions that may be more subtle but still disease related. Careful interpretation of variant data along with the above challenges of scalability and accuracy in pathogenicity prediction are further discussed by Starita et al. (2017).

Beyond experimental considerations, collective group and consortium efforts are paramount in the endeavor to create a comprehensive Atlas of Variant Effects. The coordination of work through consortiums and databases such as MaveDB can drive discovery, while avoiding redundant efforts (Esposito et al., 2019). Overall, sharing of best practices is pivotal for collective progress in the field (Azzariti et al., 2018; Denton et al., 2022; Gelman et al., 2019; Weile and Roth, 2018). Furthermore, as rare disease research has long been limited by logistical and financial barriers, developing simple and cost-effective assays for DMS will encourage greater participation among researchers. This should be considered for assays listed in Table 1 and other assays to ensure that it works in the ‘hands of many’, instead of the assay being a technical marvel that is inaccessible to most researchers. Investments in training and education are also imperative to ensure the validity, reproducibility and sustainability of DMS studies in the rare disease field. Further discussion of important considerations in enhancing the accessibility and efficiency of rare disease research can be found in existing articles (Halley et al., 2022). Integration of such efforts can facilitate a consistent march toward the goal of comprehensively understanding the clinical consequences of all variants.

## Poster

Poster
