# Supplementary figures and images for: High-throughput assays to assess variant effects on disease
Source: Dis Model Mech. 2024 Jun 28;17(6):dmm050573. doi: 10.1242/dmm.050573 (PMC11225591; doi:10.1242/dmm.050573)

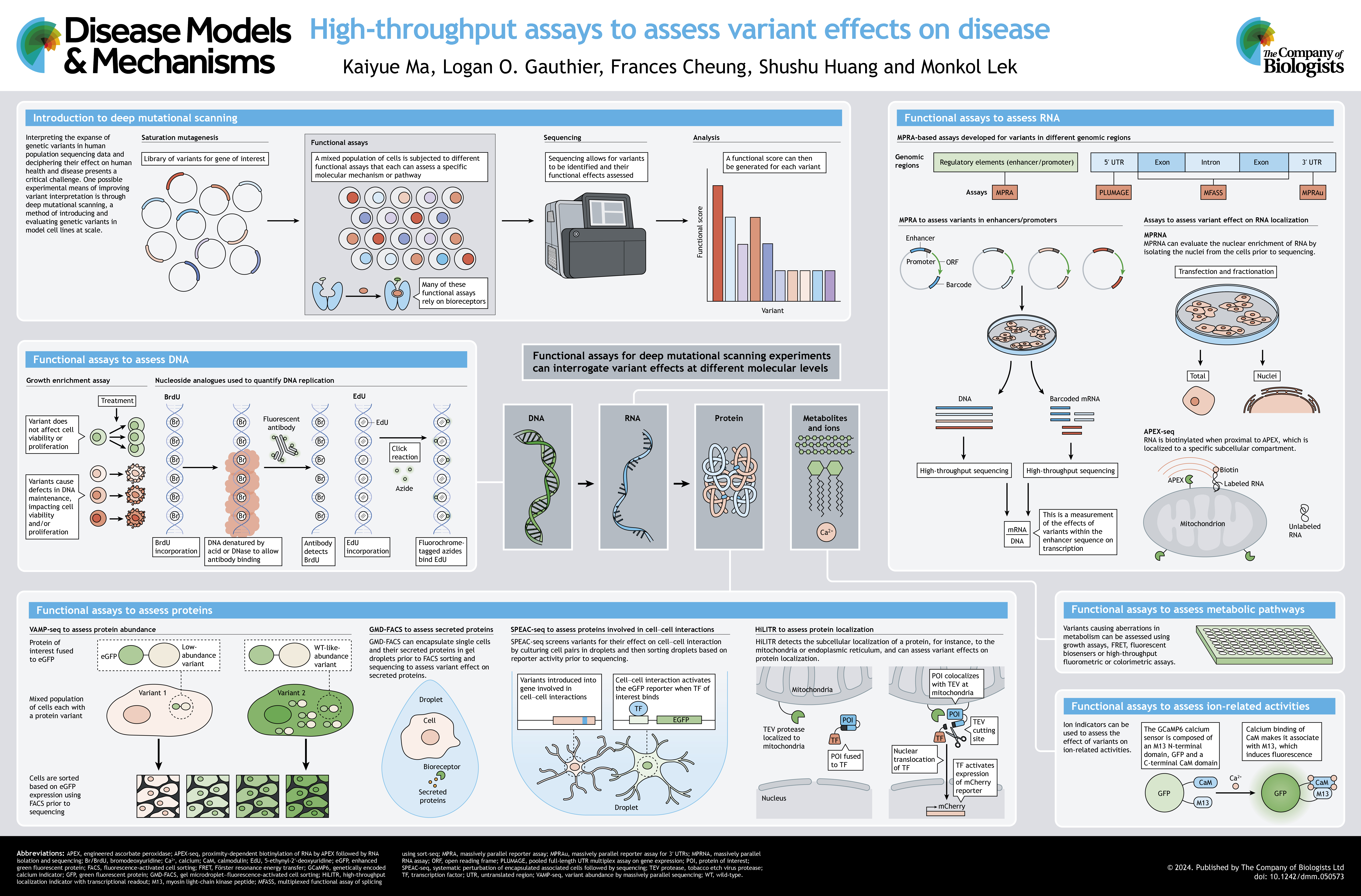

Supplement: Poster [file dmm-17-050573-s1.jpg]
